# Development and Internal Validation of a Novel Model to Identify Inflammatory Biomarkers of a Response to Escitalopram in Patients With Major Depressive Disorder

**DOI:** 10.3389/fpsyt.2021.593710

**Published:** 2021-05-20

**Authors:** Jingjing Zhou, Jia Zhou, Zuoli Sun, Lei Feng, Xuequan Zhu, Jian Yang, Gang Wang

**Affiliations:** ^1^The National Clinical Research Center for Mental Disorders & Beijing Key Laboratory of Mental Disorders, Beijing Anding Hospital, Capital Medical University, Beijing, China; ^2^Advanced Innovation Center for Human Brain Protection, Capital Medical University, Beijing, China

**Keywords:** inflammatory biomarkers, major depressive disorder, escitalopram, predictive model, followed up study

## Abstract

**Objective:** The aim of our study was to identify immune- and inflammation-related factors with clinical utility to predict the clinical efficacy of treatment for depression.

**Study Design:** This was a follow-up study. Participants who met the entry criteria were administered with escitalopram (5–10 mg/day) as an initial treatment. Self-evaluation and observer valuations were arranged at the end of weeks 0, 4, 8, and 12, with blood samples collected at baseline and during weeks 2 and 12. Multivariable logistic regression analysis was then carried out by incorporating three cytokines selected by the Least Absolute Shrinkage and Selection Operator (LASSO) regression model. Internal validation was estimated using the bootstrap method with 1,000 repetitions.

**Results:** A total of 85 patients with Major Depressive Disorder (MDD), including 62 responders and 23 non-responders, were analyzed. Monocyte chemoattractant protein-1 (MCP-1), vascular cell adhesion molecule-1 (VCAM-1), and lipocalin-2 were selected by the LASSO regression model. The area under the curve (AUC) from the logistic model was 0.811 and was confirmed as 0.7887 following bootstrapping validation.

**Conclusions:** We established and validated a good prediction model to facilitate the individualized prediction of escitalopram treatment for MDD and created a personalized approach to treatment for patients with depression.

## Introduction

Major depressive disorder (MDD) is a mental disorder, diagnosed based on self-reported symptoms and observable signs, which causes significant distress and/or functional impairment (1, 2). Effective treatments for MDD are much needed, since MDD is associated with a high cost for care as well as high morbidity and mortality (3–5). Approximately one third of all patients with depression fail to respond to conventional anti-depressant therapies (6), contributing to the global burden of the disease. Due to the current, only partially effective trial-and-error approaches are adopted for treatment selection in MDD. Predictive biomarkers that guide selection of treatment could be particularly valuable. Biomarkers could have multiple uses in psychiatry, including disease diagnosis and prediction of a therapeutic response (7). The incorporation of biomarkers into treatment of MDD could help improve efficacy of treatment and accelerate remission.

Much evidence exists regarding interactions between the brain and the immune system. Dysregulation of the immune system or inappropriate immune responses have been reported in various psychiatric disorders, particularly MDD. An aberrant inflammatory profile has been widely described for MDD and is believed to participate in the biological mechanisms involved in disease onset and response to treatment (8).

Accumulating data suggest that inflammation plays an important role in the pathophysiology of depression, and monitoring the therapeutic efficacy of drugs used to treat depression using immune parameters may identify unique patient populations (9, 10). The link between increased inflammation and depression was first reported in the early 1990s (11, 12) and led to the formulation of the macrophage hypothesis of depression (also known as the cytokine hypothesis of depression) (13, 14). Meta-analyses of the literature concluded that peripheral blood IL-1β, IL-6, TNF, and C-reactive protein (CRP) are the most reliable inflammatory biomarkers in patients with depression (10). Other factors that are associated with inflammation, such as adipokines and vascular endothelial factors, have also been shown to be involved in the pathophysiology of depression (15–17).

Regarding the potential clinical applications of the association between inflammation and depression, data indicated that inflammatory biomarkers can identify depressed patients who are less likely to respond to conventional anti-depressant treatment. Several studies have shown that anti-depressant treatments, mainly selective serotonin reuptake inhibitors (SSRIs), were associated with decreased levels of inflammatory markers (10). Different types of anti-depressant therapy may have diverse outcomes regarding changes in inflammatory cytokines (18–23). Therefore, specific associations between anti-depressant treatment and altered cytokine levels remain to be fully defined.

Immunity involves a complex interplay of multiple factors, so focusing on single inflammatory markers is likely to be inadequate. Few, if any, studies have assessed all immune- and inflammation-related factors, and whether there are specific aspects of the inflammatory response that are relevant to depression is unknown. Thus, the approach taken here, combining diverse measures of inflammation, may prove highly relevant from a clinical point of view. The least absolute shrinkage and selection operator (LASSO) method, which is a popular method for regression with high dimensional variables (i.e., genomics and proteomics) (24), was used to select the most useful predictive features from the primary data set. This is a form of regression analysis that includes a penalty for the magnitude of the regression coefficients to prevent overfitting (25). Consequently, this method is always selected to account for a large number of potentially correlated predictors (26).

The aim of our study was to identify immune- and inflammation-related factors with clinical utility for prediction of clinical efficacy in treatment of depression. We measured levels of a variety of inflammation-related markers, including cytokines, chemokines, lipocalin, vascular endothelial factor, and acute-phase reactants in plasma from clinical participants. We included as many factors as possible to identify the optimal panel of baseline inflammation-related factors that predict the anti-depressant efficacy of escitalopram. We sought immune- and inflammation-related biomarkers in depression in relation to treatment response, with the hypothesis that the anti-depressant effect of escitalopram could be predicted by baseline inflammation-related factors.

## Materials and Methods

### Patients and Study Setting

Patients with MDD were recruited from the outpatient department at of Beijing Anding Hospital, Capital Medical University. A total of 85 participants were analyzed in this study. The inclusion criteria for the study were as follows: (1) age between 18 and 65 years; (2) diagnosis of MDD by a psychiatrist using the Structured Clinical Interview for DSM-IV criteria;(3) a severity rating on the 17-item Hamilton Depression Rating Scale (HAMD-17) of ≥14 and a total score on the 16-item Quick Inventory of Depressive Symptoms–Self-Report (QIDS-SR16) that was ≥11.

The exclusion criteria were: (1) history of any clinically significant disease or laboratory abnormalities that were not stabilized or were anticipated to require treatment during the study; (2) a positive pregnancy test or breast feeding; (3) significant risk of suicide, as evidenced by scoring 3 or 4 for HAMD-17 item 3 and risk of self-harm behaviors established by the investigator; (4) alcohol or substance abuse.

The study was conducted in accordance with the Declaration of Helsinki and was approved by the Human Research Ethics Committees. All participants were free to withdraw at any time during the study. All participants signed a informed consent. Only after obtaining written informed consent from participants were study-related procedures or assessments completed.

### Study Design

Participants meeting entry criteria were administered escitalopram (5–10 mg/day) as the initial treatment (patients could reduce the dose if side effects could not be tolerated). The maximum dose of escitalopram used in the acute phase was 20 mg/day. Patients were treated by their psychiatrists at each outpatient visit and completed self-evaluation (QIDS-SR 16, FIBSER) and received observer valuations (HAMD-17) from clinicians at the end of weeks 0, 4, 8, and 12. Blood samples were collected at baseline and weeks 2 and 12 in the acute phase.

During the 14 days prior to enrolment, 10 patients with MDD were treated with escitalopram for no more than 7 days; the remaining 75 patients were not treated.

### Drug/Therapy Combination

Antipsychotics, other anti-depressants, and mood stabilizers were prohibited during the study. Use of non-benzodiazepines such as zolpidem (≤10 mg/day), zopiclone (≤7.5 mg/day), and zaleplon (≤10 mg/day) was permitted for patients with severe insomnia. Benzodiazepines such as lorazepam were permitted in patients with significant symptoms of anxiety, except for the 8 h prior to assessment. Electroconvulsive therapy, transcranial magnetic stimulation, phototherapy, electro-acupuncture, biofeedback, and vagal nerve stimulation were also prohibited. Any systematic psychotherapies (psychoanalysis, cognitive comprehension, desensitization therapy, hypnosis therapy, Morita therapy) were prohibited, but general supportive psychotherapy was allowed.

### Psychometric Assessment and Plasma Inflammatory Marker Measurements

#### Psychometric Assessment

Before each infusion, depression severity was rated using the Chinese version of the HAMD-17. According to HAMD-17 scores, responders were defined as having a 50% or greater reduction in the HAMD-17 total scores from baseline to week 12. The development of hypomanic symptoms was assessed using the Young Mania Rating Scale (YMRS). The HAMD-17 and YMRS scales were determined at baseline and weeks 4, 8, and 12. Inter-rater reliability (kappa values for categorical measures) was >0.8 for all measurements.

#### Plasma Inflammatory Marker Measurements

Peripheral blood samples were obtained by venipuncture from patients at baseline. Samples were collected into EDTA tubes and centrifuged at 2,500 rpm for 10 min at room temperature. The plasma was immediately removed, aliquoted, and stored at −80°C prior to cytokine measurements. The levels of 33 cytokines were assessed using four types of MILLIPLEX TM MAP Plex Kits (catalog number: HCYTOMAG-60K, HCVD2MAG-67K, HNDG2MAG-36K, CVD6MAG-67K; MERCK Millipore Corporation, Billerica, MA, USA) on the Luminex 200 platform (Luminex, Austin, USA) according to the manufacturer's instructions. All samples were run simultaneously for each panel and all assays were performed in duplicate. Duplicate samples from each patient were measured within one assay. All assays were carried out using a single lot number of reagents and consumables by a single operator, who was blinded to the sample sources. Data were collected using the Luminex PONENT v3.1 software and concentrations of the markers were determined using Milliplex Analyst v5.1 software.

### Statistical Analysis

Cytokines with ≥30% of missing data (values outside the ranges of detection) were excluded (27, 28). For the remaining cytokines, values below the lower detection limit (LDL) were assigned a value of half the LDL, while those above the upper detection limit (UDL) were assigned a UDL value (29–31).

Eight patients were lost to follow-up at the 12-week visit and their responses were imputed by the HAMD-17 at the 8-week visit. Continuously coded variables were reported as the mean(sd) and analyzed by *t*-test or Wilcoxon rank sum test. Categorical variables were reported as frequencies and proportions and analyzed by chi-square test. In all statistical analyses, missing data comprised <1% and were handled with the multiple imputation procedure using the R package “MICE” under the assumption that data were missing at random. Outcome information was included in the imputation model to avoid attenuation of estimated effects in later analyses (32). A formal statistical test on these variables would have to consider the scale of the experiment with type I error due to multiple comparisons. Although *P*-values are reported for these data, all information from these variables is descriptive in nature.

To build a predictive model for response using demographic and cytokine data, we used the R package “glmnet” (33) to perform the LASSO logistic regression algorithm (24, 34). This allowed us to select variables that were most predictive of a response, among all of the 26 candidate features in the data set (22 detectable cytokines, age, gender, BMI, and baseline HAMD-17 score).

A multivariable logistic regression analysis was then refitted by incorporating three cytokines (monocyte chemoattractant protein-1 (MCP-1), vascular cell adhesion molecule-1 (VCAM-1), and lipocalin-2) selected by the LASSO regression model. We assessed associations between the predictors and the outcome from resulting models using odds ratios (OR) with 95% confidence interval (CI) and *P*-value. Discrimination of the predicting model was assessed using the area under the curve (AUC) of the receiver operating characteristic and Harrell's concordance index (C-index). Calibration of the predicting model was assessed with a calibration curve and the goodness-of-fit of the model was assessed using the Hosmer-Lemeshow test (35) - *P* > 0.05 supported the goodness-of fit.

We estimated the optimism for all measures by internal validation using the bootstrap method (with 1,000 repetitions) with the relatively corrected C-index (36). Decision curve analysis (DCA) was then applied to determine the clinical relevance of the predictive model by calculating the net benefits at different threshold probabilities in the cohort (37).

All analyses were performed using R 3.5.2 (R Foundation for Statistical Computing, Vienna, Austria) and SAS (version 9.4; SAS Institute, Cary, NC).

## Results

### Patient Characteristics and Cytokine Levels

A total of 85 patients with MDD were recruited to the study (32 males, 53 females; mean age 28.95 ± 7.56 years [range 18.7–56.0]). All patients were divided into response and non-response groups (62 responders, 23 non-responders). The final dose of medicine during the follow up was higher in the non-response group (17.83 ± 3.64 mg/day) than those in the response group (15.48 ± 3.92 mg/day). All demographic and disease data in the two groups are summarized in Table 1. No differences in age, sex, BMI, onset age, illness duration, or baseline HAMD-17 scores were significant between responders and non-responders. Table 2 summarizes the levels of 22 detectable cytokines from the two groups in treatment cohort patients undergoing MDD.

**Table 1:** Demographic and clinical characteristics.

**Variables**	**MDD patients**			**T/χ2**	***p*-value**
	**Non-response *n* (%)**	**Response *n* (%)**	**All *n* (%)**		
Participants	23	62	85		
Gender^a^				0.11	0.740
Female	15(65.22)	38(61.29)	53(62.35)		
Male	8(34.78)	24(38.71)	32(37.65)		
Education^a^				3.32	0.190
Lower than Undergraduate	9(39.13)	13(20.97)	22 (25.88)		
Graduate	6(26.09)	16(25.81)	22 (25.88)		
Undergraduate	8(34.78)	33(53.23)	41 (48.24)		
Family history^a^				0.66	0.417
NO	19(82.61)	46(74.19)	65(76.47)		
YES	4(17.39)	16(25.81)	20(23.53)		
First episode^a^				0.00	0.963
NO	11(47.83)	30(48.39)	41(48.24)		
YES	12(52.17)	32(51.61)	44(51.76)		
	**Mean (SD)**	**Mean (SD)**	**Mean (SD)**		
Age (years)^c^	27.65(7.42)	29.43(7.62)	28.95 (7.56)	−1.41	0.159
Body mass index^b^	22.17(2.92)	22.87(3.63)	22.68(3.45)	−0.84	0.405
Onset age (years)^b^	23.57(6.89)	25.65(7.25)	25.08 (7.18)	−1.19	0.237
Duration of illness (years)^c^	3.04(4.12)	2.85(4.04)	2.91 (4.04)	0.23	0.817
Duration of current episode (years)^c^	0.35(0.78)	0.42(1.30)	0.40 (1.18)	0.21	0.832
*Clinical assessments*					
Baseline HAMD-17 scores^b^	19.87(4.25)	20.87(4.27)	20.60 (4.26)	−0.96	0.339
Endpoint HAMD-17 scores^c^	15.35(5.02)	4.46(3.28)	7.63 (6.28)	6.74	<0.0001
Baseline QIDS-SR scores	16.04(3.71)	14.90(3.23)	15.21 (3.38)	1.34	0.181
Endpoint QIDS-SR scores^b^	11.87(4.53)	4.88(2.78)	6.91 (4.63)	6.90	<0.0001

a *Chi-square;*

b *Independent sample t-test;*

c *Wilcoxon rank sum test*

*Body mass index is calculated as weight in kilograms divided by height in meters squared*.

**Table 2:** Inflammatory cytokine levels in treatment response and non-response groups.

**Cytokines**	**Total participants**	**Non-responders (*n* = 23)!!!break Mean (SD)**	**Responders (*n* = 62) Mean (SD)**	***p*-value**
Log of CRP	3.75(0.55)	3.68(0.52)	3.78(0.56)	0.458^a^
Sqrt of G-CSF	9.16(2.60)	8.76(2.97)	9.31(2.47)	0.390^a^
Log of Eotaxin	1.83(0.17)	1.83(0.15)	1.83(0.18)	0.993^a^
FGF2(pg/ml)	59.22(33.28)	64.34(32.31)	57.32(33.69)	0.391^a^
Log of GM-CSF	0.62(0.31)	0.62(0.31)	0.61(0.31)	0.931^a^
Log of IFNγ	0.72(0.35)	0.65(0.36)	0.75(0.35)	0.268^a^
Log of IL-1Ra	1.19(0.78)	1.39(0.76)	1.11(0.78)	0.138^a^
Log of IL-12	0.47(0.30)	0.41(0.30)	0.49(0.30)	0.280^b^
Log of IL-17	0.46(0.32)	0.40(0.33)	0.48(0.32)	0.329^a^
Log of IL-7	0.61(0.38)	0.60(0.41)	0.61(0.37)	0.980^a^
Log of IP-10	2.43(0.18)	2.38(0.17)	2.45(0.18)	0.126^b^
Lipocalin-2(ng/ml)	80.06(63.24)	105.10(116.96)	70.78(15.79)	0.530^b^
MCP-1(pg/ml)	205.99(72.00)	182.88(51.23)	214.56(76.92)	0.060^b^
Log of MCP-1β	1.25(0.41)	1.24(0.49)	1.25(0.38)	0.660^b^
Log of PDGF	3.56(0.59)	3.64(0.54)	3.53(0.61)	0.451^a^
RANTES(pg/ml)	2142.78(1097.67)	1952.99(1216.42)	2213.18(1052.10)	0.335^a^
Log of SAP	4.95(0.20)	4.90(0.17)	4.97(0.21)	0.147^a^
sCD14(ng/ml)	2396.68(585.69)	2315.97(642.28)	2426.62(565.88)	0.442^a^
sICAM-1(ng/ml)	165.57(388.68)	124.53(129.86)	180.79(448.42)	0.752^b^
TNFα(pg/ml)	9.88(6.19)	8.51(3.48)	10.39(6.89)	0.101^a^
VCAM-1(ng/ml)	661.08(140.80)	600.13(114.41)	683.70(143.73)	0.013^b^
VEGF(pg/ml)	47.86(44.73)	50.98(41.22)	46.71(46.23)	0.533^b^

a *Independent sample t-test;*

b *Wilcoxon rank sum test*.

### Feature Selection

**Figure 1:**
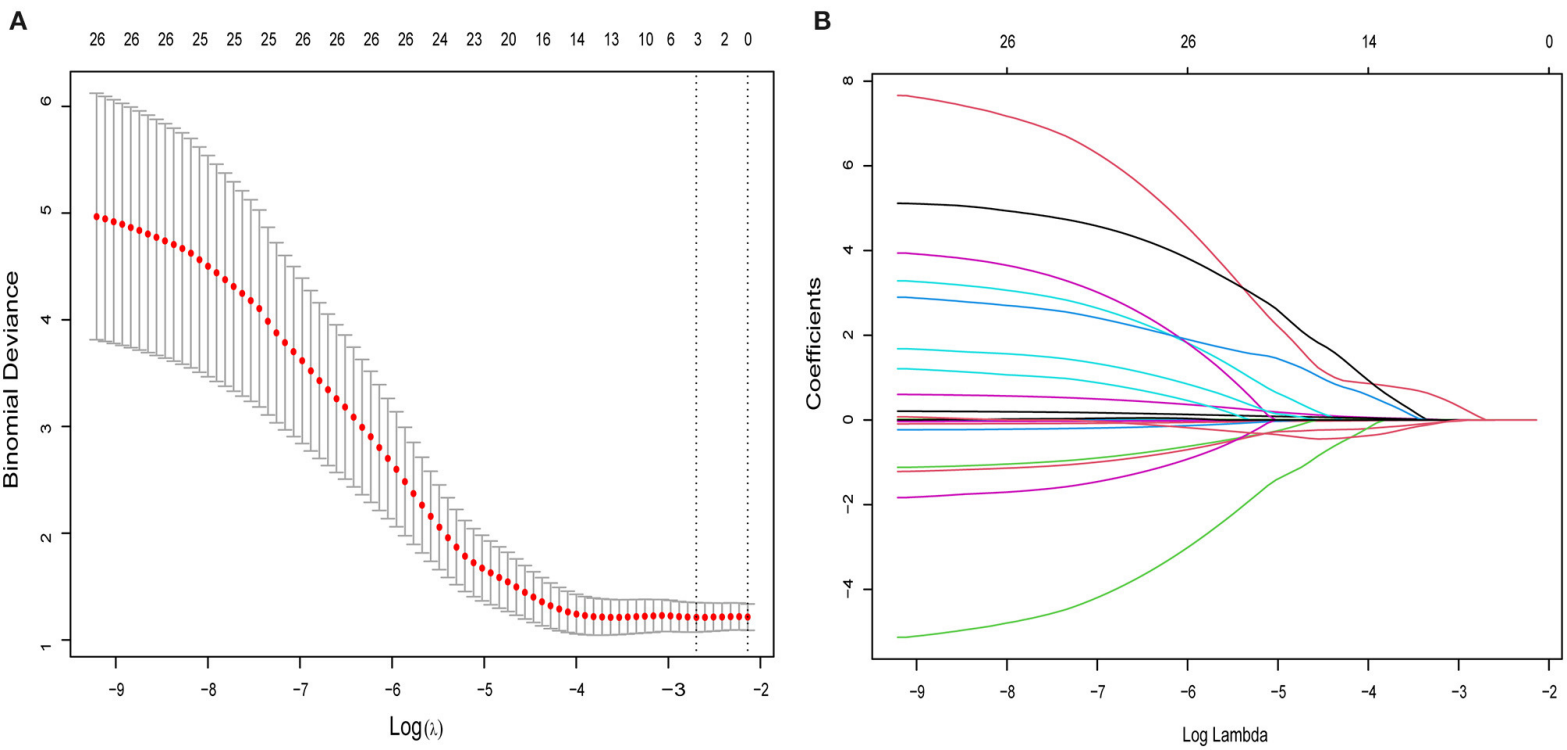
Selection of cytokines, demographic, and clinical features using the LASSO binary logistic regression model. **(A)** Optimal parameter (lambda) selection in the LASSO model using 10-fold cross-validation via minimum criteria. The partial likelihood deviance (binomial deviance) curve was plotted vs. log (lambda). Dotted vertical lines were drawn at the optimal values by using the minimum criteria and 1 SE of the minimum criteria (the 1-SE criteria). **(B)** LASSO coefficient profiles of the 26 features. A coefficient profile plot was produced against the log (lambda) sequence. A vertical line was drawn at the value selected using 5-fold cross-validation, where optimal lambda resulted in five features with non-zero coefficients. LASSO, least absolute shrinkage and selection operator.

From the demographic (age, gender, and BMI), disease (baseline HAMD-17 score), and cytokine data, 26 features were reduced to three potential predictors on the basis of the 85 patients in the cohort (~7:1 ratio; Figures 1A,B). MCP-1, VCAM-1, andlipocalin-2, had non-zero coefficients in the LASSO regression model (Table 2).

### Model Development

**Figure 2:**
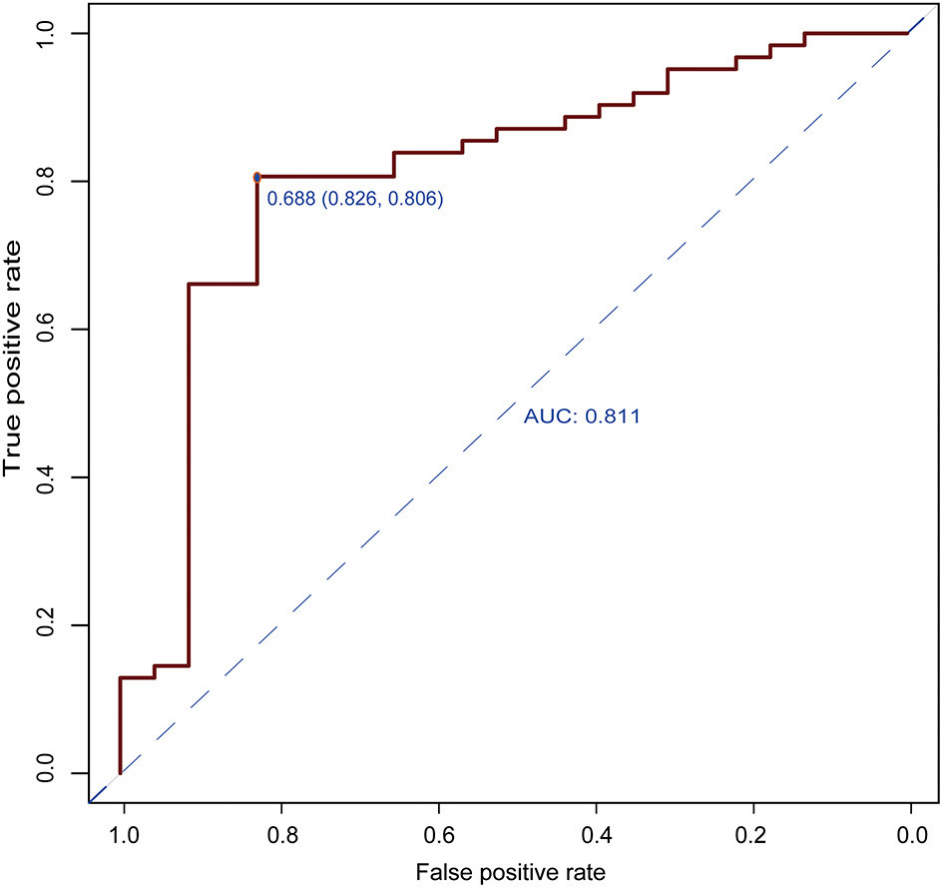
Receiver operating characteristic (ROC) curves of the predictive model. The area under the ROC curve (AUC) from the model was 0.811, the cut-off value for the prediction score at the optimum point was 0.688, the sensitivity was 82.6%, and the specificity was 80.6%.

**Figure 3:**
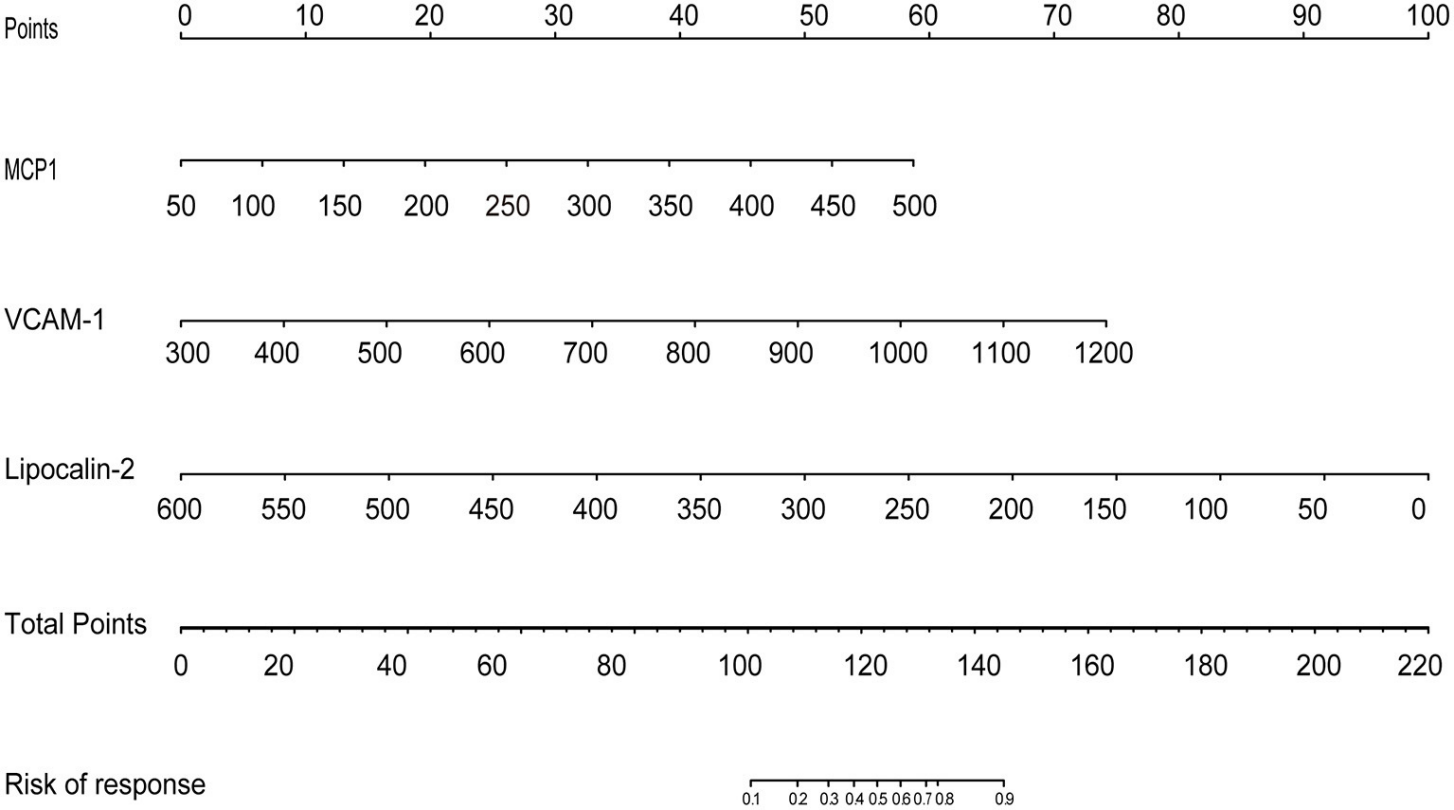
The development of a predictive nomogram for MDD response. A response predicting nomogram was developed in the study cohort that included MCP1, VCAM1, and Lipocalin. First, it is necessary to locate the patient's MCP1, VCAM1, and Lipocalin level, on the corresponding axis. The score for each value is then assigned by drawing a line upwards to the line, and the sum of the three scores is plotted on the total points line. Next, a line should be drawn straight down to identify the patient's probability of achieving a response.

The results of the multivariate logistic regression analysis for MCP-1, VCAM-1, and lipocalin-2 are presented in Table 3. A model incorporating the above independent predictors was developed and presented as a ROC curve (Figure 2) and nomogram (Figure 3). Analysis revealed that MCP-1 (OR, 1.0129; 95% CI, 1.0027–1.025), VCAM-1 (OR, 1.0082; 95% CI, 1.0031–1.014), and lipocalin-2 (OR, 0.9837; 95% CI, 0.9612–0.9972) were independently associated with treatment response. The AUC from the model was 0.811, the cut-off value for prediction score at the optimum point was 0.688, the sensitivity was 82.6% and specificity was 80.6%. The nomogram displays the multi-variant analysis effect of predictors on the risk of response at endpoint.

**Table 3:** Prediction factors for response to treatment.

**Variables**	**Prediction model**		
	**β**	**Odds ratio(95%CI)**	***P***
MCP-1	0.0129	1.0129(1.0027,1.0258)	0.02575
VCAM-1	0.0081	1.0082(1.0031,1.0142)	0.00342
Lipocalin-2	−0.0164	0.9837(0.9612,0.9972)	0.04445

*β, regression coefficient; CI, confidence interval*.

### Model Performance and Clinical Utility

**Figure 4:**
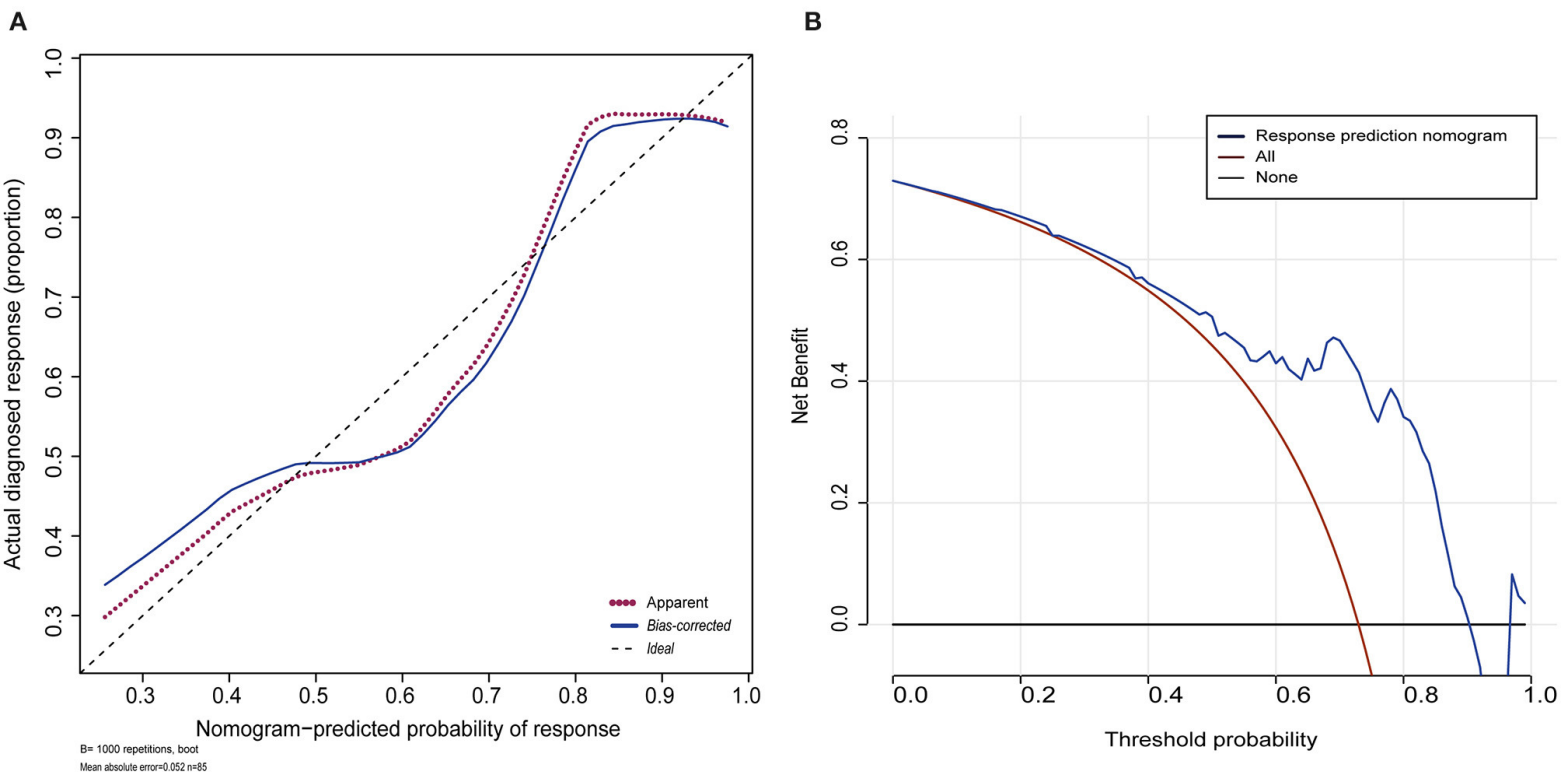
Calibration plot for the nomogram and decision curve analysis of the logistic model. **(A)** Calibration plot for the nomogram. The dotted black line indicates the location of the ideal nomogram, in which the predicted and actual probabilities are identical. The dotted red line indicates the apparent accuracy of the nomogram without correction for overfitting. The blue solid line represents the bootstrap-corrected nomogram. **(B)** Decision curve analyses (DCA) demonstrating the net benefit associated with use of the response nomogram with regards to predicting a response to treatment. The *y*-axis represents the net benefit. The red line represents the treat-all-patients scheme. The black line represents the treat-none scheme. The blue line represents the predictive nomogram scheme. The decision curve shows that if the threshold probability is 1–91%, using this nomogram in the current study to predict response adds more benefit than an intervention-in-all-patients scheme or an intervention-in-none scheme.

The shape of the curve on the calibration plots indicates that the model is well-calibrated (Figure 4A). A Hosmer and Lemeshow statistical test on the observed data for the model supported the goodness-of-fit of the model (χ2 = 13.377, *p* = 0.063). The C-index for the prediction nomogram was 0.811 (95% CI: 0.702–0.920) for the cohort and was confirmed as 0.7887 through bootstrapping validation, which suggested that the model had good discriminatory ability. In the response predicting model, apparent performance showed a good prediction capability. Figure 4B illustrates the decision curve analysis for the response predicting model. The decision curve showed that the model is useful between a threshold probability of 1 and 91%, and using this response predicting model to predict response adds more benefit to the scheme.

## Discussion

Utilization of investigative approaches coupled with multiplex immunoassay panels enables the assessment of a broad range of immune- and inflammation-related markers. In this report, we used commercially available multiplex kits to quantify a broad spectrum of inflammatory markers at baseline, in a cohort of MDD participants. Comparison of baseline factors between the 12-week treatment-responding group and the treatment-non-responding group of MDD patients revealed significant differences. Using a novel and appropriate statistical approach that simultaneously modeled dozens of sociodemographic, clinical, and inflammatory variables, we showed that inflammation-related markers at baseline can predict anti-depressant efficacy in patients with MDD. Our report describes the application of a machine learning approach to define potential inflammation-related predictors of response to the selective serotonin receptor inhibitor anti-depressant (SSRI) escitalopram, the most prescribed therapeutic drug for the treatment of depression (38), through combined LASSO and logistical regression. Three key variables (i.e., MCP-1, VCAM-1, and lipocalin-2) were identified and satisfactory performance was obtained using a parsimonious prediction model, with accuracy of 0.811.

### MCP-1

Chemokines are divided into two major families (CC and CXC) depending upon the presence or absence of an amino acid between the first two cysteines at the amino-terminal (39). Chemokines direct the cell trafficking needed to initiate T-cell-mediated immune responses and inflammation. MCP-1 is a member of the CC chemokine family and signals predominantly via the G protein-coupled CCR2 receptor. Some data point toward MCP-1 being an important mediator of the neuro inflammatory processes that take place in several neurological disorders, including autoimmune disease, obesity, and atherosclerosis. MCP-1 can affect cellular interaction, neuro modulation, and synaptic transmission, all of which are known to be altered in depression (39). In a previous study, Flaishon et al. demonstrated that CCL2 at pM levels can exert global suppressive effects on T-cell trafficking into inflamed lymph nodes. Thus, this chemokine may have clinical application as a general anti-inflammatory agent (40). The anti-inflammatory or pro-inflammatory effects of MPC-1 may be related to its specific dose.

Relatively few studies have investigated the association between MCP-1 and depression and the results of these studies are not consistent (41–44). Some reported MCP-1 levels decreased or increased after anti-depressant treatment (45, 46). In our study, MCP-1 was higher in the responding group than that in the non-responding group. The precise mechanism responsible for this association is unclear, although the neuroprotective function of neuronal chemokines (47, 48), and their ability to enhance dopaminergic activity in the central nervous system, could be possible explanations.

### VCAM-1

The expression of VCAM-1 on endothelial and other cells is induced by inflammatory stimuli and cytokines (49). Inflammation and endothelial damage are potential mechanisms that link depression with cardiovascular disease (50). A growing body of data suggest that endothelial dysfunction is associated with several clinical conditions with high cardiovascular risk, including depression (51). Symptoms of depression are related to adverse cardiovascular prognosis in patients with heart failure. Furthermore, endothelial activation and damage is characterized by increased plasma levels of soluble VCAM-1 and other factors considered to be surrogate markers of vascular disease (52).

Although the precise mechanisms involved remain unclear, some studies indicate that patients with severe depression treated with SSRIs have reduced cardiovascular risk compared to patients not receiving anti-depressant therapy (53, 54). Here, we demonstrated that VCAM-1 was significantly higher in the responding group than that in the non-responding group. Lopez-Vilchez et al. explored the potential modulating effect of anti-depressant treatment with escitalopram for 24 weeks. Their results show significant reductions in soluble VCAM-1 levels during treatment with escitalopram (55). Increased levels of soluble VCAM-1 have been reported in another study of severe depression (56), which supports the existence of endothelial damage and cardiovascular risk. However, despite this evidence, direct damaging effects on the endothelium of the humoral changes occurring in patients with depression remain poorly elucidated.

### Lipocalin-2

Lipocalin-2 (LCN2), also known as neutrophil gelatinase-associated lipocalin (NGAL), is the product of the lcn2 gene and is a glycoprotein associated with a variety of inflammatory conditions (57–59). Elisabeth et al. reported that brain lipocalin-2 may be an important biomarker of neuro-inflammation (60). As an immune-related protein, lipocalin-2 is likely to perform a dual role in the nervous and immune systems, as has been attributed to other immune-related proteins. It has been demonstrated that increased circulating levels of lipocalin-2 are significantly associated with depression in patients with heart failure (17). To our knowledge, this is the first study to evaluate the moderator effect of baseline levels of lipocalin-2 on anti-depressant treatment outcomes. NGAL can lead to reduced hippocampal neuronal growth during stress (58), which links to the “neurotrophic hypothesis of depression” (59). NGAL is therefore an interesting inflammatory component and plays an important function in the pathophysiology of depression; the precise function of NGAL in brain homeostasis warrants further investigation.

Our study provides valuable evidence that MCP-1, VCAM-1, and lipocalin-2, are putative markers of MDD due to the significant differences between pharmacological therapy responders and non-responders. Although MCP-1, VCAM-1, and lipocalin-2, were shown to be correlated with the efficacy of escitalopram in this study and were also shown to be correlated with MDD in previous studies, the interaction of these factors with MDD or antidepressant treatment, need to be investigated further.

This was a predictive study conducted to demonstrate novel methodologic approaches, such as LASSO, to the identification of predictors of recovery from depression. LASSO, an increasingly common tool in genetic research (61, 62), minimizes false discovery, and increases the generalizability of the results (62). This method of choosing predictors not only surpasses univariable analysis in terms of outcome (63), it also enables the panel of selected features to be combined into an inflammatory signature. While previous research has similarly aimed to identify models that predict treatment outcome to anti-depressant medication in MDD (64, 65), few of them focused on the response to escitalopram.

When the model was tested on a randomly selected test dataset using the bootstrapping method, its discrimination was confirmed with an accuracy of 0.7887. Calibration plot and decision curve analysis also indicated good applicability and net benefit. Currently, nomograms are widely used as prognostic devices in medicine. Given the availability of treatments in clinical settings, this approach would optimally be used to assist clinical decision-making in conjunction with response prediction models for other treatments. Our findings also provide some insight into the pathways underlying the anti-depressant effects of escitalopram, since inflammatory molecules have been implicated as potential mechanisms (66, 67). The growing trend for machine learning will hopefully create high quality evidence for the understanding of depression and drive innovations in this field. The improvement of the performance of predictive models will help to personalize treatments with safety and efficiency (68).

There are several limitations to be considered when interpreting our results. Firstly, selection bias and inadequate representation of MDD patients may have occurred, since participants were mostly included on the basis of physician referral, which was not designed to develop or evaluate a clinical decision model. Secondly, risk factor analysis did not include all potential factors that could affect escitalopram efficacy. Thirdly, although the model was tested extensively with internal validation, further external validation and replication is required. Fourthly, due to the lack of healthy controls (HCs), this study was unable to determine how these markers change relative to HCs.

In conclusion, we have proposed and validated a relatively accurate prediction model to facilitate individualized prediction of escitalopram treatment in MDD and established a personalized approach for treating patients with depression. The relationship between immune and other biological systems is complex and multifaceted. Concurrent assessment of some of the parameters involved in the inflammatory response in depression might prove useful in furthering understanding of therapeutic mechanisms. In future studies, we plan to verify the sensitivity and effectiveness of the inflammatory factor panel in efficacy prediction, using larger cohorts of patients.

## Data Availability Statement

The raw data supporting the conclusions of this article will be made available by the authors, without undue reservation.

## Ethics Statement

The studies involving human participants were reviewed and approved by Human Research and Ethics Committee of Beijing Anding Hospital (2017–24). The patients/participants provided their written informed consent to participate in this study.

## Author Contributions

JinZ: project administration and writing - original draft. JiaZ: methodology, data curation, formal analysis, and visualization. ZS: resources and investigation. LF: project administration. XZ: methodology. JY: conceptualization and writing - review and editing. GW: conceptualization. All authors contributed to the article and approved the submitted version.

## Conflict of Interest

The authors declare that the research was conducted in the absence of any commercial or financial relationships that could be construed as a potential conflict of interest.
